# Gynecological Notes

**Published:** 1894-11

**Authors:** T. Flavin

**Affiliations:** Demonstrator of Anatomy, Texas Medical College, Galveston


### GYNECOLOGICAL NOTES.

REPORTED BY T. FLAVIN, M. D.,
Demonstrator of Anatomy, Texas Medical College, Galveston.

CÆSAREAN SECTION.—Under this head Dr. J. H. Carstens, of Detroit College of Medicine, discusses the relative merits of craniotomy and Cæsarean section, where either has to be performed. He thinks no definite rule can be laid down in regard to choice, but that every case must be decided on its merits. In his own experience craniotomy is less dangerous to the mother than Cæsarean section. With the Tarnier traction forceps, craniotomy is required much less seldom than formerly. He lays down the following rule for his own use and for his students' guidance:

"If the antero-posterior diameter is three inches or more, the performance of craniotomy adds but slight additional risk to the mother over any severe forceps delivery. Hence, in cases with such a diameter, I think craniotomy is justifiable."

As an alternative and Cæsarean section, he thinks craniotomy is the proper thing where the mother has had a number of living children, and the pelvic deformity came on since; but if the woman has had a number of still-born children, or has had craniotomy performed before, and she desires a living child, Cæsarean section is the right thing. In cases out in the country, where the details of strict antisepsis are difficult or impracticable, craniotomy should be preferred, as it gives the mother the better chance for recovery.

In cases where the antero-posterior diameter is less than three inches, not only craniotomy but evisceration, and the removal of the child piecemeal would be called for, and here Cæsarean section is undoubtedly preferable.

Emphasis is laid on the point that there should be no interference or efforts at delivery made in such cases, but all preparations made for a section at the proper time.

As to the relative merits of the classical Cæsarean section and the Porro-Cæsarean section, it is stated that the former is the more dangerous and difficult, while the latter is an absolute safeguard against puerperal fever. On the other hand, subsequent pregnancy is possible after the former operation, but impossible after the latter. Here again the author thinks that the choice will depend on the circumstances of each case, and he therefore makes no rule.

---

THE MINUTE ANATOMY OF THE FALLOPIAN TUBES.—Whoever is interested in this subject will find an article thereon in the June number of *American Journal of Obstetrics and Gynecology*, by Mary A. Dixon Jones, M. D., well written, well illustrated, highly interesting and very instructive. Much painstaking histological investigation has enabled the author to bring out many new and important points connecting the anatomy and physiology of these organs. No condensation of the article would be adequate or satisfactory for lack of the illustrations, but with a view to indicating its general scope and results, the author's resume is appended:

1. In the tube walls are six layers of smooth muscles. The two main layers are the circular and the longitudinal. These interlace; the circnlar has the broader area and is nearer the lumen, the longitudinal is nearer the peritoneum.

2. The inner surface of the tube wall is made up of mynomatous or myro-fibrous connective tissue, which, in turn, is supplied with two muscle layers, a broader longitudinal and a narrow circular, both interlacing.

3. The mucosa has folds with many ramifications serving for the seclusion of the caliber during life. These folds are the result of the alternate contractions and extensions of the two muscle layers of the mucosa, the transverse and the longitudinal, which are visible throughout all the folds and all the ramifications, arranged in bundles close beneath the epithelial layer.

4. Outside of the longitudinal layer of the tube wall is the layer of blood vessels, mainly arteries and veins, in an arrangement similar to that known to exist in the wall of the uterus.

5. Beyond the vascular layers are the two narrow layers of smooth muscle fibers, both being oblique, both traceable from the uterine ostium up to the fimbriated extremity of the tube, and they correspond to the two oblique layers of the wall of the uterus. The two oblique layers are bordered outwardly by the peritoneum, and seem to serve mainly for the regulation of the afflux of blood in the subjacent arteries and veins.

6. The circular and longitudinal muscle layers are antagonistic in their action. If one layer is contracted the other is relaxed. Again, the two muscle layers of the tube wall proper are antagonistic in their action to the muscles of the mucosa. The contraction of the muscles of the tube wall is accompanied by a corresponding relaxation of the muscles of the mucosa. Within the folds the primary, secondary, and tertiary ramifications are produced by alternate contractions of smaller portions of the muscle layers of the mucosa.

